# Information Needs About Cancer Treatment, Fertility, and Pregnancy: Qualitative Descriptive Study of Reddit Threads

**DOI:** 10.2196/17771

**Published:** 2020-12-02

**Authors:** Ria Garg, Nevena Rebić, Mary A De Vera

**Affiliations:** 1 Faculty of Pharmaceutical Sciences University of British Columbia Vancouver, BC Canada; 2 Collaboration for Outcomes Research and Evaluation University of British Columbia Vancouver, BC Canada; 3 Arthritis Research Canada Richmond, BC Canada

**Keywords:** cancer treatment, health information, oncofertility, fertility, pregnancy, reproduction, social support

## Abstract

**Background:**

A reproductive health implication of the increasing incidence of cancer among women is the impact of cancer treatment on fertility.

**Objective:**

As patients are increasingly using the internet, particularly online forums, to seek and share experiences, our objective was to understand information needs about cancer treatment, fertility, and pregnancy of women with cancer as well as their caregivers.

**Methods:**

We searched threads (original posts and responses) on four subreddit sites of Reddit (“r/Cancer,” “r/TryingForABaby,” “r/BabyBumps,” and “r/Infertility”) over a 5‐year period between February 4th, 2014 and February 4th, 2019. Threads with original posts involving a lived experience or question regarding cancer treatment and female fertility and/or pregnancy or parenting/having children from the perspective of either patient or caregiver were included in our analysis. We analyzed threads using thematic analysis.

**Results:**

From 963 Reddit threads identified, 69 were analyzed, including 56 with original posts by women with cancer and 13 with original posts by caregivers. From threads made by patients, we identified themes on becoming a part of an online community, impacts of cancer treatment and fertility concerns on self and social relationships, making family planning decisions, and experiences with medical team. We also identified a theme on the impact of cancer treatment and fertility concerns on caregivers.

**Conclusions:**

Reddit provided a rich pool of data for analyzing the information needs of women facing cancer. Our findings demonstrate the far-reaching impacts of cancer treatment and fertility on physical, mental, and psychosocial health for both patients and their caregivers.

## Introduction

There is need to better support women with cancer as they deal with cancer treatment and impacts on fertility [1], from clinical aspects such as fertility preservation to psychosocial aspects spanning psychological, social, behavioural, and ethical considerations [2]. This is important, as treatments used in cancer (including chemotherapy, radiotherapy, and surgery) may affect fertility by impairing reproductive and endocrine functions [1,3]. Of particular interest is health information needs, which arise when individuals perceive gaps in their knowledge regarding a specific health-related topic [4]. In 2016, Benedict et al [5] surveyed 346 women who had completed cancer treatment at a single center about their fertility information needs and found that up to 62% reported unmet information across topics queried such as risk of infertility, risk of early menopause, and options to preserve their fertility. Although it is important to understand information needs based on questions queried by researchers, drawing this information from patients without prompts is also necessary to identify other areas of priority. A potential source of patient-centered information is social media and online forums [6]. As more patients access these mediums to seek accounts of personal experiences from others navigating similar issues, so forms a valuable, naturally generated pool of data for examining patients’ information needs, which has yet to be fully utilized [7]. Thus, our aim was to conduct a qualitative descriptive study of threads on the social news website, Reddit. We sought to address the following research question: what are the information needs regarding cancer treatment, fertility, and pregnancy of women diagnosed with cancer and/or their caregivers/partners.

## Methods

### Study Design and Data Source

We conducted a qualitative descriptive study of online discussions, using data gathered from Reddit. Here, submitted content is organized according to subreddits on specific subjects (“r/subject”). Users (“Redditors”) can subscribe to subreddits and participate in conversations (“threads”) by either starting an original thread or commenting on other users’ threads. Reddit offers a large and variable platform for information gathering, the site has over 430 million monthly active users, averages 21 billion screen views per month, and ranks as the 5^th^ most visited website in the US as of December 23^rd^, 2019 [8]. Reddit is composed of user-generated content, allowing users to share media, follow one another, and share anecdotal information in the form of personal experiences. Redditors can share publicly accessible content as anonymity is provided with the use of pseudonyms and usernames [9]. Users can freely share personal experiences and engage in open and honest discussion without feeling restricted, a barrier present on other social media platforms such as Facebook, which mandate individuals accessing the website to use their real names [10].

### Search Strategy

Our strategy aimed to identify threads over a 5-year period between February 4^th^, 2014 and February 4^th^, 2019 through a systematic approach to searching subreddits and the application of specific inclusion and exclusion criteria. Given that there is no specific subreddit for oncofertility, we searched four relevant subreddits: r/Cancer, r/BabyBumps, r/Tryingforababy, and r/Infertility. Threads were gathered from each subreddit by searching relevant terms or words. For example, given that the subject matter of r/Cancer subreddit related to the maternal disease of interest, terms included the following: fertility, infertility, menopause, pregnancy, and pregnant. Conversely, for the 3 subreddits related to reproductive health, terms included the following: cancer, chemotherapy, and radiation. At this stage, selected threads included an original post and at least one comment/response.

We then reviewed the original post to apply the following inclusion criteria: indicating having a diagnosis of cancer *and* having received or may receive gonadotoxic treatment (eg, chemotherapy, radiotherapy) *and* sharing a lived experience, concern, or information need regarding female fertility or pregnancy. We also considered threads where the original post was shared by a caregiver of an individual with cancer who had received or may receive gonadotoxic treatment. For our purposes, we defined caregiver as any individual providing support to a woman with a cancer diagnosis. The search results are summarized in Multimedia Appendix 1. The application of the inclusion criteria to threads was conducted by two authors (RG and NR) and discrepancies were discussed and resolved.

### Data Extraction

**Figure 1:**
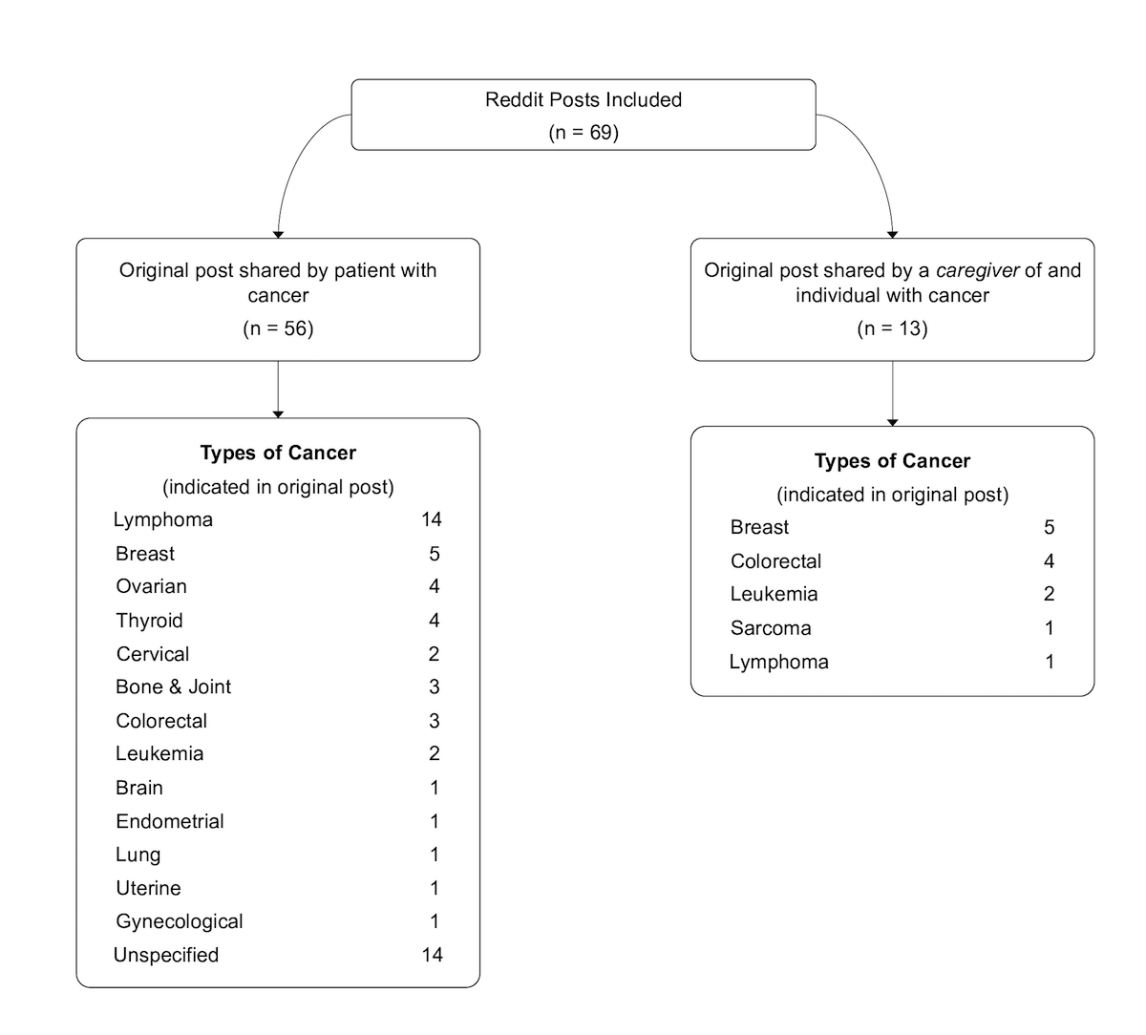
Types of cancer indicated in original posts of included Reddit threads.

We downloaded threads meeting inclusion criteria as portable document files. We extracted the following information: author of the original thread (ie, patient with cancer or caregiver), average thread length (ie, the number of comments/replies to the original post), average number of unique users participating in each thread. Where possible, we also extracted information on the type of cancer and cancer treatment(s), as outlined in Figure 1.

### Qualitative Analysis

We conducted two separate thematic analyses, the first for threads with original posts by patients with cancer and the second for threads with original posts by caregivers. Thematic analysis is a form of descriptive qualitative analysis applied to all final threads (original post and comments) included in the study to review and interpret narrative data through the identification of themes [11,12]. The value of a thematic study design is its ability to formulate a deeper understanding of the studied population’s viewpoint, actions, and relationships [11,12]. We used de novo line by line coding where every line in the transcript is used to formulate emerging ideas. These ideas were transcribed into codes or a short phrase representing key attributes of narrative information. Following, codes were sorted and organized to identify patterns and formulate subcategories. Themes were named and defined based on these results. We used NVivo 12 (QSR International) for all analyses.

### Ethics

According to the University of British Columbia’s Behavioral Research Ethics Board, given our use of publicly available information from a social network site (ie, one that does not require an account or password to access content) and the fact that we did not use privately sourced data, this research is exempt form review.

## Results

### Search Results

Altogether, we downloaded 963 threads across 4 subreddits, “r/Cancer,” “r/Tryingforababy,” “r/BabyBumps,” and “r/Infertility” between February 4^th^, 2014 and February 4^th^, 2019. Overall, 69 reddit threads were included in our analysis. For 56 threads, the original post was shared by a patient with cancer, and for 13, the original post was shared by a caregiver. All original posts were by unique individuals as assessed by their usernames. Table 1 summarizes information on included threads.

**Table 1:** Summary of Reddit threads.

Author of original post, Subreddit		Subscribers, n	Threads^a^ downloaded, n	Threads included, n	Average thread length^b^, n	Average number of unique users, n
**Patient with cancer**						
	r/Cancer	24700	214	17	8.2	5.2
	r/Tryingforababy	32000	200	8	9.9	6.9
	r/BabyBumps	117000	275	12	27.8	17
	r/Infertility	13000	274	19	15	9.5
**Caregiver**						
	r/Cancer	24700	214	12	10.8	6.6
	r/BabyBumps	117000	275	1	5	4

^a^Between February 4^th^, 2014 and February 4^th^, 2019.

^b^Average length of thread is defined as the number of comments/replies to the original post.

### Qualitative Results

Thematic analysis of threads where the original post was shared by a patient with cancer resulted in 5 themes: 1) becoming a part of an online community; 2) impact of cancer treatment and fertility concerns on self; 3) impact of cancer treatment and fertility concerns on social relationships; 4) making family planning decisions; and 5) experiences with medical team. Thematic analysis of threads where the original post was shared by a caregiver resulted in an additional theme on 6) impact of cancer treatment and fertility concerns on the caregiver. These themes, corresponding conceptual categories, and representative quotations are described as follows. To provide context to quotations, we included information on subreddit, type of cancer, and cancer treatment, where available. Additional representative quotations for identified themes are available in Multimedia Appendix 2.

### Theme 1: Becoming a Part of an Online Community

Women diagnosed with cancer often have questions or concerns regarding the impact of cancer treatment on their state of fertility leading them to seek a community for social support. In our study, we found that women used Reddit to join an online community for reasons captured by the following 4 conceptual categories.

#### Subtheme 1: Connecting With Individuals Facing Similar Circumstances

Many women expressed a desire to or gratitude for connecting with individuals who have faced similar circumstances, reporting feeling isolated among family and friends who are unable to understand their experiences. Statements of gratefulness such as, “This community has provided me with comfort through, knowing there are others that can completely relate to me” (r/Infertility; unspecified cancer; chemotherapy), highlight the capacity for an online community to provide support and help users feel that they are not alone. Others expressed difficulty finding a community they identified with prior to discovering Reddit, as one user shared, “I was under the impression that people like me just didn't exist!” (r/BabyBumps; bone and joint cancer; chemotherapy, radiotherapy, and surgery). Women valued Reddit’s online community, expressing gratitude for having the opportunity to connect with others, as one user stated, “I'm in tears realizing that you all are so kind” (r/Infertility; lymphoma; radiotherapy), when describing the positive impact other Redditors have had on her life.

#### Subtheme 2: Seeking Advice or Information From Others Online

Women often turn to Reddit’s online communities to pose questions and ask for information regarding cancer treatment and fertility. Many users asked others to share their personal experiences to help them anticipate future challenges, as one user stated, “I’m looking for any information about what others may have experienced and what I should expect” (r/Cancer; leukemia; chemotherapy). Aside from information, women also sought advice to aid their decision-making. For example, prior to undergoing chemotherapy, one user shared how she felt overwhelmed by the uncertain state of her fertility and was seeking advice for how best to cope, stating, “I feel helpless. What do I do now?” (r/Cancer; bone and joint cancer; chemotherapy).

#### Subtheme 3: Sharing Personal Victories

Online discussions have given women a platform for documenting their triumphs and sharing positive testimonials. Many users shared their experiences of having children after cancer treatment, expressing how fortunate they felt after overcoming the obstacles they faced in their journey to conceive. One user highlighted the steps she took postchemotherapy to preserve fertility despite low chances of natural conception and shared the joy she felt once she was able to conceive, stating, “Our little man is truly a miracle baby” (r/BabyBumps; ovarian cancer; chemotherapy, surgery). Other women shared personal victories in the form of remaining determined to conceive despite facing challenges with fertility. One user shared remaining hopeful for her future childbearing ability despite undergoing systemic cancer treatment, stating, “Multiple health issues going against chances of my pregnancy but HOPE IS ON!” (r/Infertility; colorectal cancer; chemotherapy, radiotherapy, surgery).

#### Subtheme 4: Providing Social Support

Participation in online communities allowed users to provide social support, defined as actions that encourage or help another’s journey to having children. Users would respond to others with statements such as, “First, congrats on beating cancer! That must not have been easy. I hope IVF is successful” (r/Infertility; breast cancer; chemotherapy), capturing congratulatory and encouraging sentiments. Users also provided information by sharing personal accounts of their experiences with fertility post cancer treatment, describing steps they had taken to conceive, or directing others to resources.

### Theme 2: Impact of Cancer Treatment and Fertility Concerns on Self

Cancer treatments and fertility concerns may affect an individual in many ways, as outlined by 2 conceptual categories.

#### Subtheme 1: Coping With Physical Impacts of Cancer Treatments

Cancer treatments, such as radiotherapy and chemotherapy, may have systemic health impacts. Users used Reddit forums to discuss symptoms of premature menopause after cancer treatment, with many expressing difficulty coping with the physical impacts. They shared how certain symptoms may remain persistent even after cancer treatment has been stopped. As one user stated, “It has been over a year since my last treatment and I am still having them (hot flashes) regularly” (r/Cancer; cancer unspecified; chemotherapy). Users reported experiencing a broad spectrum of physical symptoms post cancer treatment, stating, “I had some nerve damage which is irreversible. Its not just dryness, the skin changes and deteriorates” (r/Cancer; leukemia; chemotherapy).

#### Subtheme 2: Navigating Fertility Concerns

Users shared feelings of emotional distress; many found processing all the information regarding cancer treatment and its adverse effects on future fertility to be overwhelming. One user described navigating cancer treatment decisions as “the start of a nightmare I never imagined I would be in” (r/Cancer; sarcoma; chemotherapy) due to the lack of clarity regarding cancer treatment impacts on future fertility. Many women struggled with disentangling their post cancer treatment symptoms from premature menopause, which may occur after systemic cancer treatment. One user stated, “I know early menopause is a very real possibility with chemotherapy but I'm only 25. Is it possible that these (symptoms) are just temporary” (r/Cancer; lymphoma; chemotherapy). Women who experienced cancer treatment induced infertility shared stories of struggle to move past their cancer diagnosis and feeling as if their choice to conceive in the future was taken away from them. One user stated, “I'm losing my potential child before I ever had a chance to realize I wanted him” (r/Cancer; sarcoma; chemotherapy).

### Theme 3: Impact of Cancer Treatment and Fertility Concerns on Social Relationships

The impacts of cancer treatment and fertility concerns on individuals’ social relationships is outlined by 2 conceptual categories.

#### Subtheme 1: Shaping Intimate Partner Relationships

Cancer treatment and fertility concerns may impact a woman’s perception of their partners, defined as the individual who has chosen to share their life with the women with cancer. Many women described internalizing their struggles with conception and blaming themselves for not providing their partner with a child. One user shared feeling sad and guilty after undergoing cancer treatment, stating, “If he had only fallen in love with some other girl, he wouldn’t have had to deal with all of this” (r/Cancer; Ovarian; Chemotherapy). Users also shared experiencing strained partner relationships due to a perceived lack of understanding or inability to appropriately acknowledge their fertility concerns and struggles with conception. One user shared, “when I mention I’m upset about losing my fertility and he (my husband) tries to tell me it isn’t as important as my life. Well no shit, but I can still be upset about it” (r/Cancer; gynecological; chemoradiation).

#### Subtheme 2: Navigating Changing Self-Identity

An individual’s changing self-identity related to cancer treatment and its impacts on fertility may also impact their social relationships. Many women wrote about alienating themselves from their family and friends, as they feared discussing the topic of fertility. Some shared feeling resentment towards others with children, stating, “I'll either see baby posts on Facebook or come across women with a child in public, and feel so bitter and angry towards them” (r/Infertility; lung cancer; chemotherapy, surgery), and described surrounding themselves with friends and family who were able to conceive as a reminder of their own struggles. Others reported feeling frustrated that their inability to conceive was oversimplified and misunderstood. One user expressed feeling pressured to conceive quickly from her mother-in-law, stating, “I'm pretty sure (she) thinks her son married a defective uterus” (r/Infertility; unspecified; chemotherapy).

### Theme 4: Making Family Planning Decisions

Prior to receiving cancer treatment, women are required to make several important decisions related to future fertility and family planning. The questions and concerns women may come across while making family planning decisions are outlined by 2 conceptual categories.

#### Subtheme 1: Perspectives Regarding Fertility Treatments

Prophylactic fertility preservation treatments, such as egg freezing, need to occur prior to receiving cancer therapy; as such, a women’s cancer severity, prognosis, and desire for children in the future may influence her decision to pursue fertility treatment. Some women reported feeling uncomfortable delaying cancer treatment to pursue fertility treatment due to the risk of cancer reoccurrence or progression, stating, “the idea of waiting while this really aggressive tumor grew inside of me was...unsettling” (r/Infertility; breast cancer; chemotherapy). Others believed they might regret not attempting fertility preservation and shared either a desire to receive treatment or gratitude for having already received prophylactic fertility treatment. Many women also struggled with uncertainty when deciding if they should pursue fertility preservation. One user shared, “How do I know if I will regret not having kids?? Right now it sounds ok, but in 10 years” (r/Infertility; lymphoma; chemotherapy), struggling to decide whether she should delay chemotherapy to pursue fertility treatment.

#### Subtheme 2: Financial Concerns

Decisions about fertility treatment and family planning may also be influenced by an individual’s financial state. Several women shared concerns about pursuing fertility treatments due to financial constraints, as one user stated, “IVF (in vitro fertilization) is a long ways a way. The cancer basically depleted our savings” (r/TryingForABaby; unspecified; chemotherapy). Others reported becoming skeptical of the benefits of fertility treatments, such as in vitro fertilization, after investing thousands of dollars into medical treatments without achieving favorable outcomes. As one user stated, “Sunken cost fallacy is something I think of all the time. Especially with our frozens (eggs) we still have” (r/Infertility; lymphoma; chemotherapy).

### Theme 5: Experiences With Medical Team

When receiving cancer or fertility treatments, women require assistance from their medical team, which may include an oncologist, gynaecologist, obstetrician, and reproductive endocrinologist. However, several Reddit users expressed challenges with their medical team as outlined by 2 conceptual categories.

#### Subtheme 1: Seeking Doctor With Relevant Patient Experience

Many users felt their doctors did not focus enough on their state of fertility during and after cancer treatment, leading them to seek doctors who have experience working with premenopausal cancer patients with fertility concerns. Several users shared the positive impact a physician with relevant patient care experience had on their fertility journey. When describing how her endocrinologist tailored treatments to her reproductive needs, one user stated, “experience with cancer patients is a must” (r/Infertility; ovarian cancer; chemotherapy, surgery). Another user stated, “Everyone there was ready to call it quits and wrote me off” (r/Infertility; unspecified; chemotherapy), sharing her struggles with fertility treatment until she connected with a more experienced endocrinologist who was able to offer her further treatment options and support her desire to conceive.

#### Subtheme 2: Feeling Lack of Support

Many women shared incidences of miscommunication regarding the impact of cancer treatment on their fertility with their medical team. Several women articulated a lack of focus on fertility preservation during cancer treatment. One user said, “I feel like my doctor heard my history, saw my test results and has basically written me off” (r/Infertility; lymphoma; radiation), feeling her desire to protect her fertility was dismissed as secondary to her cancer diagnosis. Others felt frustrated, having to constantly self-advocate for their fertility concerns to be addressed by their medical team. One user described the high level of persistence she exercised for her medical team to consider her fertility status, stating, “Why do I have to be the one to take initiative?” (r/Tryingforababy; cancer unspecified; chemotherapy). Some felt disheartened about their ability to conceive after their doctor expressed discouraging views about their fertility post cancer treatment, as one user stated, “the dr came in and said he thinks it is best if I stop trying (to conceive). That was the end of that” (r/Infertility; lymphoma; chemotherapy, radiotherapy, surgery).

### Theme 6: Impact of Cancer Treatment and Fertility Concerns on the Caregiver

Caregivers of cancer patients, including partners, family members, and friends, may also be impacted by their loved one’s cancer treatment and fertility concerns. The caregivers’ involvement in their loved one’s cancer treatment is outlined by the 2 following conceptual categories.

#### Subtheme 1: Searching for Avenues of Support for Cancer Patient

Caregivers of cancer patients turned to Reddit in search of avenues of social support for their loved ones. Many inquired how they could support their loved ones. One user stated, “I want to send her something to show support, but I'm not sure what would be best?” (r/Cancer; breast cancer; chemotherapy). Caregivers sought information regarding cancer treatment and impacts on fertility to remain informed about the possible treatments and likely outcomes available for their loved ones, such as one user who asked, “In this circumstance, is there any possibility of having a child at this point (post cancer treatment) through fertility treatments and specialists?” (r/Cancer; colorectal cancer; radiotherapy, chemotherapy).

#### Subtheme 2: Coping With Cancer Treatment and Fertility Concerns

Caregivers of cancer patients may also experience emotional distress regarding cancer treatment and fertility concerns. Many users expressed feeling stressed, worried, and anxious watching their loved one struggle with uncertainty related to disease prognosis and fertility, sharing, “I know my family is experiencing emotions all too painfully familiar to everyone here” (r/Cancer; sarcoma; chemotherapy). Some intimate partners expressed their frustrations concerning their inability to have children, as one user shared, “Would have been nice to know that possibility (infertility) before she went through radiation but radiologist never mentioned it” (r/Cancer; colorectal cancer; radiotherapy, surgery). However, others worried their emotions may be perceived as resentment by their partner with cancer. After his wife experienced chemotherapy induced menopause, one user shared, “I cannot convince her that this is the case. She thinks I'm angry, that I blame her and her ‘defective body’ (her words)” (r/Cancer; colorectal cancer; radiotherapy, chemotherapy), using Reddit to process his own emotions.

### Thematic Map Describing Relationships Between Themes

**Figure 2:**
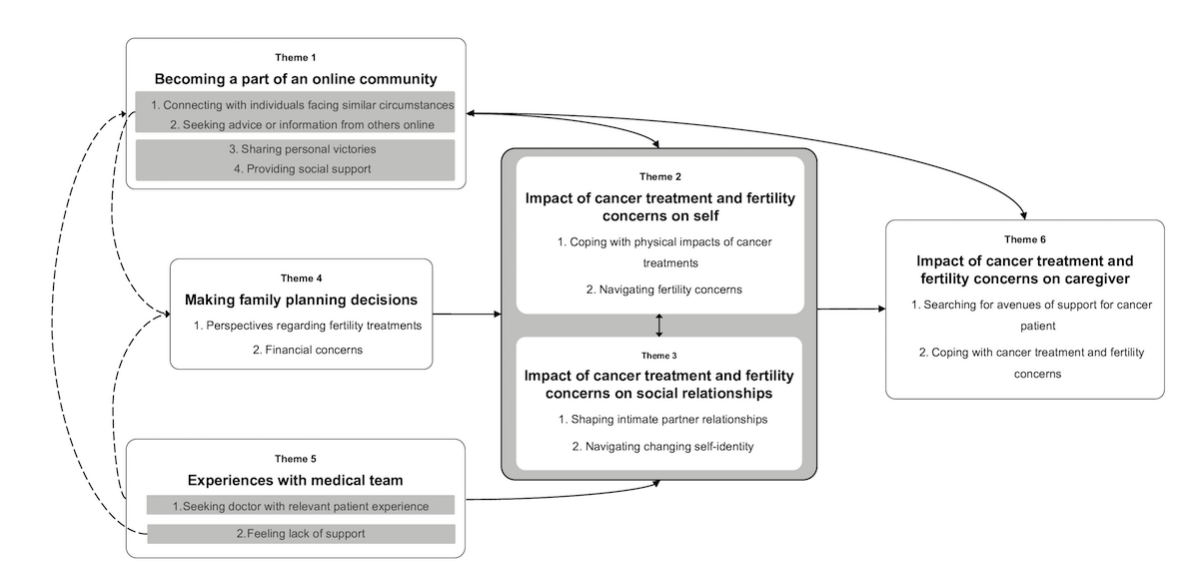
Thematic map of identified themes. Solid arrows depict relationships between themes, while dashed arrows depict relationships between categories across and within themes.

The thematic map in Figure 2 outlines the interrelationships between the 6 identified themes. Experiencing cancer treatment and fertility concerns may affect women in various areas of their life, leading to impacts on self (Theme 2) which may also impact their social relationships (Theme 3), and vice versa. As women cope with the impacts of cancer treatment and fertility concerns, they may turn to Reddit to seek or share information and connect with others facing similar circumstances, leading them to join an online community (Theme 1). A woman’s medical team is an integral component of her cancer treatment experience (Theme 5); therefore, her experiences with such a team may further contribute to the impact of cancer treatment and fertility concerns on self and social relationships (Theme 2 and 3). If women feel a lack of support from their medical team, this may lead them to seek support from other sources, such as Reddit’s online community (Theme 1). In addition to their medical team (Theme 5), as women connect with others online and seek advice (Theme 1). This may also influence their family planning decisions (Theme 4), which overall contributes to the impact of cancer treatment on the patient and their social relationships (Theme 2 and 3). Finally, caregivers are also impacted by cancer treatment and fertility concerns (Theme 6), leading to them seek an online community (Theme 1).

## Discussion

Using a systematic approach to searching Reddit threads and applying thematic analysis to those that met our study inclusion criteria, we identified themes that captured the information needs of women with cancer regarding cancer treatment and fertility that led them to this online community. Furthermore, since we also considered Reddit threads initiated by caregivers of women with cancer, we identified an additional theme regarding the impact of cancer treatment and fertility concerns on the caregiver. Key findings include the far-reaching impacts of cancer treatment and effects of fertility issues on physical and psychosocial health for women with cancer and the perceived lack of support, particularly from their medical team. The impacts on personal relationships must also be acknowledged, from both perspectives of women with cancer and their caregivers. Altogether, our study findings have implications for highlighting ongoing challenges in oncofertility and the need for better support for women with cancer, particularly when addressing their concerns and information needs regarding cancer treatment and fertility.

Our study adds to the body of work on the information needs of women with cancer regarding cancer treatment and fertility concerns by systematically searching and applying qualitative research methods to publicly available threads on the popular and widely accessed social news website, Reddit. Prior qualitative studies have used more traditional methods of interviews and focus groups and have largely focused on women with breast cancer in single centers. In 2003, Thewes et al [13] explored the fertility and menopause related information needs of young women with a diagnosis of breast cancer. Participants discussed psychosocial impacts of unmet information, identified a desire for receiving fertility related information, and reported a discord between the perceived importance of fertility among doctors and cancer patients [13]. Additionally, a 2004 study by Partridge et al [14] investigated the concerns experienced by women diagnosed with breast cancer and reported that 73% of participants expressed some degree of concern regarding fertility. They also explored factors that may influence women as they make cancer treatment decisions, such as age, desire for more children, or prior difficulty conceiving [14]. In comparison to the abovementioned studies, our study offers a broader perspective on the impacts of undergoing cancer treatment and experiencing fertility concerns, as they may not be isolated events in a woman’s life. Our findings demonstrate how cancer therapy and fertility concerns affect both the individual’s physical and psychosocial health, their social relationships, and interactions with their healthcare team, as well as how these factors may influence their decision making. Notably, our study also identified the impact of financial considerations when making family planning decisions and the role of online mediums for sharing information, connecting with others, and finding avenues of social support.

Given our use of publicly available online data through Reddit, our study adds to the body of literature describing the role of the internet as a source of information regarding cancer treatment and fertility. A recent 2019 study by Brochu et al [15] surveyed women (n = 313) and men (n = 254) seen in Canadian fertility and urology clinics to assess their use of internet-based resources for accessing infertility-related information and support. The authors reported that a greater majority of participants sought information from the internet about fertility than from noninternet sources (87.8% vs 12.2%, respectively) and noted that women in particular were significantly more likely to use the internet to search for both medical information and patient experiences shared online related to cancer therapy and infertility [15]. Indeed, the findings of this survey reflected the themes elucidated in our study, particularly theme 1, which describes how women with cancer used Reddit to join an online community through connecting with others facing similar circumstances, seeking advice, sharing personal experiences, and providing support.

Additionally, a noteworthy finding of our study is the perceived lack of support from medical teams captured in Theme 5, particularly regarding fertility information needs. This is consistent with a prior systematic review by Logan et al [16], which stated that cancer patients place great importance on their oncofertility care and have unmet support needs. Interestingly, though threads mentioned oncologists, gynaecologists, obstetricians, and reproductive endocrinologists, we did not note mentions of allied healthcare providers who may also have roles in providing education and support to women with cancer regarding cancer treatment and fertility. A recent scoping review by Anazodo et al [17] on oncofertility services in cancer care identified several key domains required for appropriate medical and psychological oncofertility provision, including providing quality information about fertility risk and preservation options to patients, timely service provision, and age-appropriate care before, during, and post cancer treatment and further developed competency framework for developing such services and training staff. Additionally, in order to facilitate service provision, The authors noted the importance of establishing referral pathways, defining the role and scope of practice of all involved health care providers, improving communication amongst the patient’s healthcare team, and ensuring all members of the healthcare team have received adequate oncofertility training. Of particular note is the potential to specifically target oncofertility training towards allied health professionals (eg, nurses, social workers, psychologists, and physician assistants) who maintain direct and long-term relationships with cancer patients. Although work in this field is limited, one pilot study suggests that allied health professional are interested in and perceived a benefit from receiving expanded training in discussing reproductive health concerns with cancer patients [18].

Finally, unique to our study is the consideration of perspectives from caregivers of women with cancer, which elucidated a theme that highlights that they, too, are affected by cancer treatment and impacts on fertility, emotionally and psychosocially. In their systematic review and meta-synthesis, LeSeure and Chongkham-ang [19] highlighted the importance of caregiving, particularly in cancer, over the past decade and discussed recommendations for continued research involving such individuals. The desire for caregivers to remain involved in their loved one’s cancer journey is also supported by our findings, as caregivers used Reddit to inquire about the impacts of cancer treatment on fertility. Overall, our study highlights a need for supporting caregivers as they may also be impacted by cancer treatment and fertility concerns.

Strengths and limitations of our study warrant discussion. We demonstrated a systematic approach to identifying and applying qualitative research methodology to eligible subreddit threads to elucidate themes pertaining to information needs of women with cancer regarding cancer treatment and fertility. Although such an approach is gaining popularity in other areas, particularly mental health and rheumatology [20] (including a recent study by our research team [7]), it has not been widely used in psycho-oncology research and, to our knowledge, there is only one prior study [21] applying content analysis on Reddit threads specifically regarding cancer, which has been published as an abstract. Properties of Reddit, including use of pseudonyms that provide users anonymity and unrestricted word count for thread entries, may contribute to greater authenticity of discussions, which may not be applicable to other social media sites such as Facebook [22-25]. However, there is potential selection bias with respect to individuals who may be more likely to use Reddit, including those who have access to the internet. Furthermore, the anonymity leads to lack of demographic and disease information about users. Nonetheless, we were able to extract information on type of cancer diagnosed for the majority of included threads from the patient arm (42 of 56 posts) and caregiver arm (13 of 13 posts) of the study. Furthermore, we were also able to extract information on the type of cancer treatment. Although the self-reported nature may be a limitation, given the specificity of a cancer diagnosis, we anticipate a high likelihood that original posts are written by individuals experiencing the impacts of the disease and its treatment on fertility. Finally, although we identified themes that touch on fertility-related issues for women with cancer and the perceived lack of support, particularly from their medical team, use of Reddit threads as our data source precluded ability to further probe into these issues.

In conclusion, women with cancer receiving gonadotoxic treatment are among the many patients who consult internet resources to ask questions and seek information about fertility and reproductive health. We demonstrated that online communities, specifically Reddit, provide a naturally generated data source for understanding information needs of these patients. Findings on the far-reaching impacts of cancer treatment and fertility on physical, mental, and psychosocial health for both patients and their caregivers and perceived lack of support from medical teams speak to a need for implementing multifaceted approaches for support.

## Appendix

Multimedia Appendix 1 Results of systematic review performed to identify Reddit threads regarding cancer and fertility concerns.

Multimedia Appendix 2 Illustrative quotes for themes on information needs and concerns posted on Reddit forums about fertility and cancer.
