# Supplementary figures and images for: Information Needs About Cancer Treatment, Fertility, and Pregnancy: Qualitative Descriptive Study of Reddit Threads
Source: JMIR Cancer. 2020 Dec 2;6(2):e17771. doi: 10.2196/17771 (PMC7744261; doi:10.2196/17771)

Multimedia Appendix 1

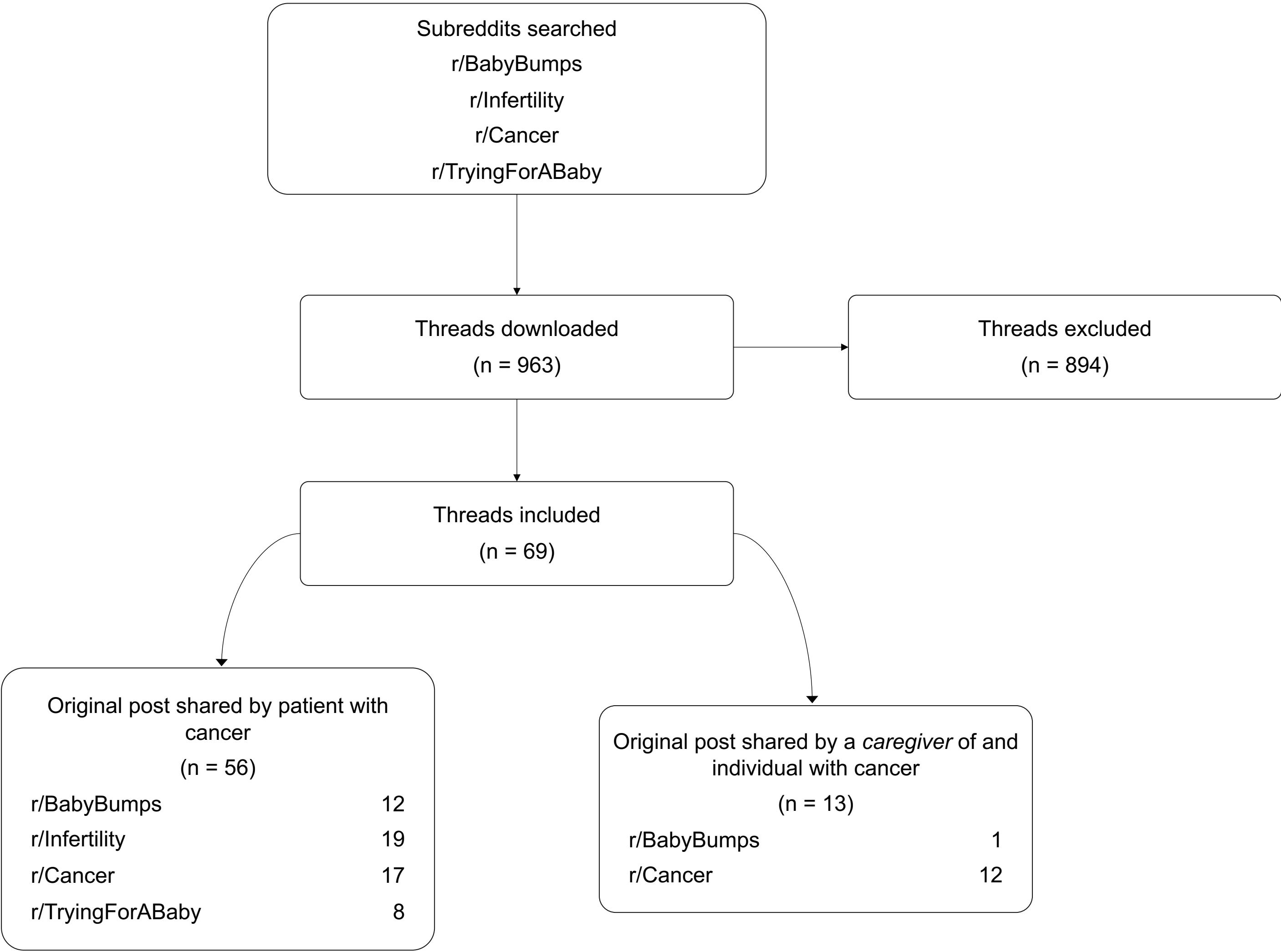

Supplement: Multimedia Appendix 1 [file cancer_v6i2e17771_app1.pdf]
