# Supplementary material for: Information Needs About Cancer Treatment, Fertility, and Pregnancy: Qualitative Descriptive Study of Reddit Threads
Source: JMIR Cancer. 2020 Dec 2;6(2):e17771. doi: 10.2196/17771 (PMC7744261; doi:10.2196/17771)
Supplement: Multimedia Appendix 2 [file cancer_v6i2e17771_app2.pdf]

Multimedia Appendix 2

Theme 1: Becoming part of an online community	
Connecting with individuals facing similar circumstances	*"But I'm wondering if anyone has gone through something similar and if you had issues conceiving or any issues with your pregnancy"* **(r/Babybumps; Lymphoma; Chemotherapy)**<br><br>*"Thank you so much!! I can't believe no one has told me about this before or that this test hasn't come up when I've researched in the past. Thanks again!"* **(r/Cancer; Bone + Joint; Surgery)**
Seeking advice or information from others online	*"Anyone have a successful pregnancy after chemo?"* **(r/BabyBumps; Lymphoma; Chemotherapy)**<br><br>*"I am new to all of this. Does anyone have input on the cost of egg retrieval, storage, IVF and surrogacy?"* **(r/Infertility; Ovarian; Chemotherapy, Surgery)**
Sharing personal victories	*"We didn't expect it to work, but neither of us wanted to start any course of fertility treatments until we knew for sure that we couldn't do it on our own. To our amazement, we got our positive pregnancy test result in November. I'm 26 weeks pregnant and our baby is absolutely perfect"* **(r/BabyBumps; Leukemia; Chemotherapy)**<br><br>*"I AM CANCER FREE. I have been cancer free for about a month, and I have started getting ready to try. We are not going to tell any family members, just in case. I know the. Chemo may have messed with my ability to get pregnant. But yay! I am back."* **(r/TryingForABaby; Lymphoma; Chemotherapy)**
Providing social support	*"I would recommend asking your oncologist if they could refer you to a reproductive health specialist/gynecologist familiar with the issue."* **(r/Cancer; Leukemia; Chemotherapy)**

	"So in my experience I started by asking my oncologist about conception and if it was safe. Once she approved it I immediately set up an appointment with a RE because I didn't want to waste a year trying if I wasn't fertile" **(r/Infertility; Lymphoma; Radiotherapy)**
Theme 2: Impact of cancer treatment and fertility concerns on self	
Coping with physical impacts of cancer treatments	*"The big issue for me was vaginal atrophy. Yup, it's just as nice as it sounds! I was so preoccupied with the cancer treatment that by the time I realised what was going on with my foof I had some nerve damage which is irreversible. Its not just dryness, the skin changes and deteriorates."* **(r/Cancer; Leukemia; Chemotherapy)**<br><br>*"But recently, I've been getting lots of mood swings, really dry skin, and hot flashes"* **(r/Infertility; Lung; Chemotherapy, Surgery)**
Navigating fertility concerns	*"it is frustrating! Sometimes it feels like my body has betrayed me or something."* **(r/Babybumps; Bone + Joint; Chemotherapy, Radiotherapy, Surgery)**<br><br>*"I liked the idea of getting closure about all this so I didn't wonder about what if years down the road like I always had before. So I tentatively agreed to continue treatment, mostly with the intention of having closure of the issue altogether"* **(r/Infertility; Unspecified; Chemotherapy, Radiotherapy)**
Theme 3: Impact of cancer treatment and fertility concerns on social relationships	
Shaping intimate partner relationships	*"My fiance* is supportive, but I'd be a naive fool not to reserve a little piece of me, just in case it's all too much for him, just in case he can't accept a wife whose ovaries have been poisoned into infertility. He didn't offer me that ring with the expectation that this would happen."* **(r/Cancer; Sarcoma; Chemotherapy)**<br><br>*"I have fallen in love with an amazing guy. So wonderful that for the first time in my life I became sad I wouldn't ever*

	be able to give him the ultimate gift, a baby that was a little bit of me and a little of him." **(r/Infertility; Unspecified; Chemotherapy, Radiotherapy)**
Navigating changing self-identity	*"To ask her to take care of me while I'm literally dying in front of her, I would never in a thousand years wish that on her, and now I'm forcing it on her because neither of us has a choice"* **(r/Cancer; Sarcoma; Chemotherapy)**<br><br>*"Since starting our TTC journey, 4 couples who started around the same time are now pregnant. It's been hard to congratulate them without feeling like a failure each time."* **(r/Tryingforababy; Thyroid; Radiotherapy)**
Theme 4: Making family planning decisions	
Perspectives regarding fertility treatments	*"The egg retrieval is really what I'm leaning towards at this point, because I can really see me kicking myself in 10 or 15 years if I don't. It sounds like kind of a rough process from what I've seen here and been told of, but maybe the best choice in this situation"* **(r/Infertility; Lymphoma; Chemotherapy)**<br><br>*"My oncologists decided I needed to be induced at 32 weeks (of pregnancy) so that I could have a PET scan to determine whether the tumours in my abdomen and neck where still active. I was pretty upset, we had discussed the option of waiting until I was at least 36 weeks"* **(r/BabyBumps; Lymphoma; Chemotherapy)**
Financial concerns	*"Which on all the scales, shows menopause. As for treatments, I can not afford them, and have state insurance."* **(r/Tryingforababy; Unspecified; Chemotherapy)**<br><br>*"I should have at minimum 5 chemoradiation cycles because the size of the primary tumor before surgery. That would render me infertile and we don't have the money for egg freezing."* **(r/Cancer; Gynecological; Chemotherapy, Radiotherapy)**

Theme 5: Experiences with medical team	
Seeking doctor with relevant patient experience	*"I then switched doctors to see a more experienced oncologist in Miami, he was able to give me a few more options than I had with the first doctor. Since then I have undergone 2 surgeries in an attempt to eradicate the cancer and preserve my fertility"* **(r/Infertility; Cervical; Chemotherapy, Surgery)**<br><br>*"I am also hoping that along the way I will speak to some sort of GI surgical specialist with cancer survivor experience who can tell me with certainty that we can do the egg retrieval without risk of scratching the bowel."* **(r/infertility; Unspecified; Chemotherapy, Radiotherapy)**
Feeling lack of support	*"my RE does not communicate with me at all. I haven't seen or spoken to her once since our initial consult and exam."* **(r/Infertility; Unspecified; Chemotherapy)**<br><br>*"i am just so frustrated that there was no steps taken to make sure I would be able to conceive."* **(r/Tryingforababy; Unspecified; Chemotherapy)**
Theme 6: Impact of cancer treatment and fertility concerns on caregiver	
Searching for avenues of support for cancer patient	*"I'm just wondering from people who have been through it, is there anything your partner did that helped? Anything that I can do other than be supportive when she starts, or before she starts?"* **(r/Cancer; Lymphoma; Chemotherapy)**<br><br>*"What are some good resources so I can learn more about everything without it becoming too overwhelming?"* **(r/Cancer; Bone + Joint; Chemotherapy)**
Coping with cancer treatment and infertility	*"doctor just told her straight that she won't be able to have children after the treatment, because of the effects of radiation on the uterus. My GF and I were in shock and asked for alternatives"* **(r/Cancer; Colorectal; Chemotherapy, Radiotherapy, Surgery)**<br><br>*"A rough week of transfer, waiting anxiety--long entry"* **(r/Cancer; Leukemia; Chemotherapy, Surgery)**
